# Reverse game: from Nash equilibrium to network structure, number and probability of occurrence

**DOI:** 10.1098/rsos.241928

**Published:** 2025-05-21

**Authors:** Ali Ebrahimi, Mehdi Sadeghi

**Affiliations:** ^1^ School of Biological Sciences, Institute for Research in Fundamental Sciences (IPM), Tehran, Iran; ^2^ National Institute for Genetic Engineering and Biotechnology (NIGEB), Tehran, Iran

**Keywords:** game theory, network, reverse game, Nash equilibrium

## Abstract

In this paper, we introduce a reverse game approach to network-modelled games to determine the network structure among players that can achieve a desired Nash equilibrium. We consider three types of network games: the majority game, the minority game and the best-shot public goods game. For any proposed Nash equilibrium, we identify the conditions and constraints of the network structure necessary to achieve that equilibrium in each game. Acceptable networks—i.e. networks that satisfy the assumed Nash equilibrium—are not unique, and their numbers grow exponentially based on the number of players and the combination of strategies. We provide mathematical relationships to calculate the exact number of networks that can create the specified Nash equilibrium in the best-shot public goods game. Additionally, in the majority and minority games, the relationships presented under special conditions specify the number of networks. We also investigate the distribution of acceptable networks as microsystems associated with the existing Nash equilibrium and their probability of occurrence. Our simulations indicate that the distribution of acceptable networks according to density follows a normal distribution, and their probability of occurrence increases. In other words, denser networks are more likely to lead to the desired Nash equilibrium.

## 1. Introduction

The decisions players make are often shaped by the individuals they interact with, which can be represented as a network. In recent years, network theory has developed to model different phenomena using complex networks, considering components as vertices and the connections between them as edges [1–7]. This approach facilitates a comprehensive analysis of existing linear, nonlinear and multivariate relationships.

Game theory is a mathematical framework that investigates how rational players—such as humans, genes, companies and countries—make decisions in interactive situations, whether through conflict or cooperation. It serves to analyse player interactions within networks, where behaviour is influenced by neighbouring players, as each individual’s payoff depends on both the actions of their neighbours and the structure of the complex network [8–16].

A key question is what decisions players should make to maximize their utility based on the network structure. Nash equilibrium, a fundamental concept in game theory, assumes that no player benefits from a change in strategy, given that the strategies of other players remain constant. The set of strategies derived from Nash equilibrium is utilized to identify solutions for players in different issues [17–22]. Multiple Nash equilibria can be generated for a finite game, and many attempts have been made to find the Nash equilibrium in different network games [23–32].

The behaviour of individuals within a network can be forecasted through various methods, depending on the type of game and the strategies employed by the players. These methods can ultimately uncover the behaviour patterns of each player in the network, as well as the collective behaviour influenced by the network structure. On the other hand, numerous studies highlight how dynamic networks can evolve based on players’ strategies and interactions. For example, Pacheco *et al*. proposed a model of active linking, illustrating how individuals can pursue new interactions, thereby affecting Nash equilibria through the evolution of strategies and network topologies [33,34]. Traulsen *et al*. examined how local interactions in structured populations result in unique emergent behaviours compared with well-mixed populations [35]. Perc & Szolnoki emphasized the importance of considering both strategy evolution and network structure [36]. Liu *et al*. studied the influence of community structures on coevolutionary dynamics [37], while Wu *et al*. demonstrated that network topology impacts the probability of achieving pure Nash equilibria [38]. Yang *et al*. investigated adaptive linking in networked games and its effects on outcomes [39]. Lastly, Berner *et al*. offered a thorough review of adaptive dynamical networks, summarizing recent developments in understanding how network dynamics influence game theory outcomes [40].

We investigate the inverse of this problem: given the behaviour or actions of players in a network at equilibrium, what potential network structures could drive this behaviour? Can we determine the network structure that resulted in these actions at equilibrium? Is this structure unique, or can multiple structures produce the same outcome? Are all these networks equally likely to exist if we assume a Nash equilibrium that reflects the macroscopic state of the players’ strategies?

We highlight that specific structures can facilitate the achievement of the desired Nash equilibrium. Consequently, the dynamics of the network—where communication among individuals and their strategies evolve simultaneously—are essential. From this perspective, our study aligns with the aforementioned articles. However, rather than focusing on the dynamic processes discussed in those works, we identify acceptable structures based on the conditions and constraints inherent to each game. This approach is significant because it allows us to categorize all possible structures into two distinct groups: acceptable networks and unacceptable networks.

We consider three games: the majority game, the best-shot public goods game and the minority game. These games have been modelled and analysed through networks in various studies. For example, Karotkin developed a network based on weighted majority rules and the weighted majority game [41]. Mukherjee *et al*. applied the weighted majority game to select fog devices designed to minimize delay and energy consumption in both indoor and outdoor settings [42]. Eksin & Paarporn examined which players to control and how to manage them to achieve desirable outcomes in learning dynamics through network-based game modelling [43]. Liu *et al*. employed multi-layer networks to investigate the coevolution of agents’ behaviours and noise parameters in a majority vote game [44]. Lo *et al*. offered a theoretical framework for networked minority games based on strategy pattern dynamics [45]. Jiang *et al*. explored the multiple effects of social influence on cooperation in interdependent network games [46]. In another study, modal logic was presented for reasoning about strategies in social network games [47]. Fagiolo & Valente studied a localized version of the minority game, where agents are positioned on the nodes of a directed graph [48]. The dynamics of cooperation in minority games within alliance networks have also been investigated [49]. Another study examined the dynamics of networked evolutionary minority games on complex networks [50]. Kirley modelled evolutionary minority games using small-world networks [51]. Jin *et al*. applied evolutionary game theory to present the K-hop evolutionary best-shot networked public goods game aimed at increasing social welfare [52]. Papadimitriou & Peng explored public goods games on directed networks [53]. Another study computed equilibria in binary networked public goods games [54]. Pichler & Shapiro researched public goods games on adaptive coevolutionary networks [55], while another work investigated stochastic stability in best-shot network games [56].

In line with the reverse game approach, we seek to identify the networks that can achieve the desired outcome and realize the specified Nash equilibrium. Assuming that a Nash equilibrium reflects the macroscopic state of players’ strategies, are all possible networks equally likely to exist? In the considered games, our objective is to pinpoint the networks capable of achieving the desired outcome and realizing the specified Nash equilibrium. Our simulations reveal that, for none of the three games, the probability of occurrence of acceptable networks follows a uniform distribution. Instead, the likelihood of network occurrence increases with density, indicating that denser networks are more likely to achieve the desired Nash equilibrium.

## 2. Results

The present study considered the network games with *n* players, in which each player must adopt one of two strategies 0 and 1.


(2.1) $$V=\left\{1,2,\ldots ,n\right\}\;\;\;\;\;\;and\;\;\;\forall i\in V\;;\;A_{i}=\left\{0,1\right\}.$$

In this case, the total number of possible combinations of players’ strategies is equal to $2^{n}$. Assuming each of these combinations as a hypothetical Nash equilibrium, the network G with vertices V and edges E was specified to indicate how players should connect within the network, with each player’s neighbours determined by E. In other words,


(2.2) $$\forall i\in V\;;\;\;\;N_{i}\left(G\right)=\left\{\;j\;|\left(i,j\right)\in E\right\}.$$

In network games, a player’s decisions are not affected by the strategies of all players; instead, their effectiveness is determined by the network edges. Consequently, each player’s choices are influenced solely by the strategies of their neighbours. When vertices are considered similar, only the number of neighbours who have adopted each possible strategy impacts the strategy selection of each vertex. Thus, within this framework, in Nash equilibrium, the strategies of both vertices will be identical if the number and strategies of their neighbours are equivalent. Based on this understanding, we have established a set of rules and restrictions for each of the three games—the majority game, the best-shot public goods game and the minority game—that the network among players must satisfy to correspond with the existing Nash equilibrium, as described below.

In each game, an acceptable network, based on the assumed Nash equilibrium, is defined as one where the conditions and constraints of the game are satisfied for all players, thereby leading to the assumed Nash equilibrium. In majority game, the majority of each player’s neighbours must adopt the same strategy as the player. In minority game, the majority of each player’s neighbours must choose a different strategy from that of the player. In best-shot public goods game, each player using strategy 0 must have at least one neighbour with strategy 1, and there should be no connections between players using strategy 1. Thus, an acceptable network meets these specific conditions for all players based on their respective strategies.

### 2.1. Majority game

In the majority game, each player prefers to choose the strategy that most of its neighbours have chosen. Specifically, for each player i if the number of members in the set $N_{i}(G)$ who have selected strategy 1 exceeds those who have chosen strategy 0, then player i will benefit more by adopting strategy 1 instead of strategy 0, and vice versa. Let $a_{i}$ represent the strategy of player i, and let $a_{N_{i}\left(G\right)}$ denote the combination of strategies among the neighbouring vertices of player i. Then


(2.3) $$\left\{ \begin{array}{l} u_{i}\left(1,a_{N_{i}\left(G\right)}\right)>u_{i}\left(0,a_{N_{i}\left(G\right)}\right)\;\;\;\;\;\;\;\;\;\;\;\;\;\;if\;\;\;\;\;\;\;\;\;\;\;\;\;\;\;\;\frac{\sum_{j\in N_{i}\left(G\right)}a_{j}}{|N_{i}\left(G\right)|}>\frac{1}{2}\\ u_{i}\left(1,a_{N_{i}\left(G\right)}\right)<u_{i}\left(0,a_{N_{i}\left(G\right)}\right)\;\;\;\;\;\;\;\;\;\;\;\;\;\;if\;\;\;\;\;\;\;\;\;\;\;\;\;\;\;\;\frac{\sum_{j\in N_{i}\left(G\right)}a_{j}}{|N_{i}\left(G\right)|}<\frac{1}{2} \end{array}\right..$$

Assuming a hypothetical Nash equilibrium that reflects the macroscopic state of the players’ strategies, let $n_{0}$ and $n_{1}$ represent the number of players adopting strategies 0 and 1, respectively, such that $n_{0}+n_{1}=n$. If vertex i adopts strategy 1 in this equilibrium, then the following relationship holds in the corresponding network:


(2.4) $$\begin{array}{r} |N_{i}\left(G\right)\cap V_{1}|\;>\;|N_{i}\left(G\right)\cap V_{0}| \end{array},$$

where $V_{0}=\left\{v\in V;\;a_{v}=0\right\}\;\text{and}\;V_{1}=\left\{v\in V;\;a_{v}=1\right\}.$

In other words, the number of edges from vertex i to the set of vertices $V_{1}$ must always exceed the number of edges from vertex i to the set of vertices $V_{0}$. Additionally, for every vertex j that adopts strategy 0, the following relationship must be satisfied:


(2.5) $$|N_{j}\left(G\right)\cap V_{1}|<|N_{j}\left(G\right)\cap V_{0}|.$$

If these conditions are not met for even a single vertex in the network, the assumed Nash equilibrium cannot be established. Therefore, in the basic state, we can consider a network that for each vertex i adopts strategy 1 and for each vertex j with strategy 0,


(2.6) $$|N_{j}\left(G\right)\cap V_{0}\left|>0\;\text{and}|N_{i}\left(G\right)\cap V_{1}\right|>0\;\text{and}\;|N_{i}\left(G\right)\cap V_{0}\left|=|N_{j}\left(G\right)\cap V_{1}\right|=0.$$

Whenever an edge in the network leads to an increase in the counts of ${|N}_{i}\left(G\right)\cap V_{0}|$ and/or ${|N}_{j}\left(G\right)\cap V_{1}|$, additional edges should be added to ensure that the counts of ${|N}_{i}\left(G\right)\cap V_{1}|$ and${|N}_{j}\left(G\right)\cap V_{0}|$ also increase. This should be done in a manner that maintains the inequalities stated in relations (2.4) and (2.5).

### 2.2. Best-shot public goods game

In the Nash equilibrium of the best-shot public goods game, if all neighbours of vertex i adopt strategy 0 (i.e. ${|N}_{i}\left(G\right)\cap V_{1}|=0)$, then vertex i must adopt strategy 1. Conversely, if at least one neighbour of vertex i adopts strategy 1 (i.e. ${|N}_{i}\left(G\right)\cap V_{1}|\neq 0)$, then vertex i must adopt strategy 0. In other words, if 0<c<1, the following relationship holds:


(2.7) $$u_{i}\left(a,G\right)=\left\{ \begin{array}{l} \;\;\;\;\;\;\;\;\;\;\;\;\;\;\;\;\;\;\;1-c\;\;\;\;\;if\;\;\;\;a_{i}=1\\ 1\;\;\;\;\;\;\;\;if\;a_{i}=0\;and\;\exists \;j\in N_{i}\left(G\right)\;s.t.\;\;a_{j}=1\\ 0\;\;\;\;\;\;\;\;if\;a_{i}=0\;and\;\forall \;j\in N_{i}\left(G\right)\;\;\;a_{j}=0 \end{array}\right..$$

Therefore, there should be no edges connecting the vertices within the set $V_{1}$, and each vertex in the set $V_{0}$ must have at least one neighbour in the set $V_{1}$. The presence or absence of an edge between the vertices in the set $V_{0}$ is irrelevant.

### 2.3. Minority game

The following relationship holds in the minority game:


(2.8) $$\left\{ \begin{array}{l} u_{i}\left(1,a_{N_{i}\left(G\right)}\right)<u_{i}\left(0,a_{N_{i}\left(G\right)}\right)\;\;\;\;\;\;\;\;\;\;\;\;\;\;\;if\;\;\;\;\;\;\;\;\;\;\;\;\;\;\;\frac{\sum_{j\in N_{i}\left(G\right)}a_{j}}{|N_{i}\left(G\right)|}>\frac{1}{2}\\ u_{i}\left(1,a_{N_{i}\left(G\right)}\right)>u_{i}\left(0,a_{N_{i}\left(G\right)}\right)\;\;\;\;\;\;\;\;\;\;\;\;\;\;\;if\;\;\;\;\;\;\;\;\;\;\;\;\;\;\;\frac{\sum_{j\in N_{i}\left(G\right)}a_{j}}{|N_{i}\left(G\right)|}<\frac{1}{2} \end{array}\right..$$

Thus, for each vertex i with strategy 1 and each vertex j with strategy 0, the following conditions must hold:


(2.9) $$|N_{i}\left(G\right)\cap V_{1}|<|N_{i}\left(G\right)\cap V_{0}|\;and\;|N_{j}\left(G\right)\cap V_{0}\left|<|N_{j}\left(G\right)\cap V_{1}\right|.$$

Therefore, in the basic state, we can consider a network where


(2.10) $$|N_{i}\left(G\right)\cap V_{0}\left|>0\;\text{and}\;|N_{j}\left(G\right)\cap V_{1}\right|>0\;\text{and}\;|N_{i}\left(G\right)\cap V_{1}\left|=|N_{j}\left(G\right)\cap V_{0}\right|=0.$$

Whenever an edge in the network leads to an increase in the counts of ${|N}_{i}\left(G\right)\cap V_{1}|$ and/or ${|N}_{j}\left(G\right)\cap V_{0}|$, additional edges should be added to ensure that the counts of ${|N}_{i}\left(G\right)\cap V_{0}|$ and ${|N}_{j}\left(G\right)\cap V_{1}|$also increase in such a way that the inequalities stated in relation (2.9) are consistently maintained.

**Figure 1.**
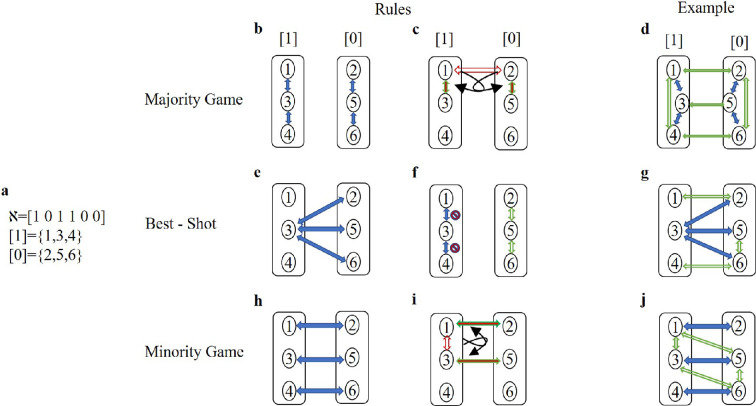
The reverse game: from Nash equilibrium to acceptable structures. (a) A hypothetical Nash equilibrium for the *six-player* game, where three players adopt strategy 1 and three players adopt strategy 0. (b–d) Majority game. (b) In the basic state, the number of neighbours adopting different strategies is considered to be 0, while at least one neighbour adopts the same strategy. (c) If the presence of an edge in the network leads to an increase in the number of neighbours with different strategies, additional edges should be added to ensure an increase in the number of neighbours with the same strategy. (d) An acceptable network configuration in the majority game. (e–g) Best-shot public goods game. (e) Every vertex with strategy 0 must have at least one neighbour with strategy 1. (f) There should be no edges connecting vertices within the set of vertices that adopt strategy 1. The presence or absence of edges between vertices with strategy 0 is irrelevant. (g) An acceptable network configuration in the best-shot public goods game. (h–j) Minority game. (h) In the basic state, at least one neighbour adopts a different strategy, while the number of neighbours adopting the same strategy is considered to be 0. (i) If an edge in the network results in an increase in the number of neighbours with the same strategy, additional edges should be added to ensure an increase in the number of neighbours with different strategies. (j) An acceptable network configuration in the minority game.

In figure 1, a Nash equilibrium for the six*-*player game is illustrated, where three players {1, 3, 4} adopt strategy 1 and three players {2, 5, 6} adopt strategy 0. For each of the three games mentioned above, the conditions and restrictions governing the network among the players are presented, along with examples of acceptable networks.

### 2.4. The number of acceptable networks

Based on the proposed conditions for acceptable networks, it is clear that these networks are not unique to each game corresponding to a given Nash equilibrium; rather, they depend on the number of players and the combination of strategies. To calculate the total number of acceptable networks associated with a specific Nash equilibrium, we present the following mathematical formulae. In the best-shot public goods game, theorem 1 specifies the number of networks for any desired distribution of players adopting strategies 0 and 1. In the majority and minority games, theorems 2 and 3 determine the number of acceptable networks under certain conditions. These theorems will be proven in §4.

In our analysis, each vertex in the network represents a player, and the strategy of each player is defined within the context of the assumed Nash equilibrium. When determining the number of acceptable networks for this equilibrium, it is vital to consider the number of labelled graphs. This is important because isomorphic structures can exist where corresponding vertices in two graphs may adopt different strategies, and this distinction must be acknowledged.

**Theorem 1**. *The number of networks with n nodes that* $\left|V_{0}\right|=n_{0}$ *and* $\left|V_{1}\right|=n_{1}$ *(i.e.* $n_{0}+n_{1}=n$*), in the best-shot public goods game is determined by the following equation*:


(2.11) F1(n0,n1)=2n0(n0−1)2×∑i=0n0(−1)i×(n0i)×2(n0−i)×n1.

**Theorem 2**. *If* $F_{2}\left(n_{0},n_{1}\right)$*represents the number of networks that exist in the Nash equilibrium of the majority game, then the following propositions apply*:


(2.12)a)F2(n0,0)=∑i=0n0(−1)i×(n0i)×2(n0−i2)(2.13)b)F2(n0,n1)=F2(n1,n0)(2.14)c)F2(n0,2)=F2(n0,1)=F2(n0,0).

**Theorem 3**. *If* $F_{3}\left(n_{0},n_{1}\right)$ *denotes the number of networks that exist in the Nash equilibrium of the minority game, then the following propositions apply*:


(2.15)a)F3(n0,0)=F3(n0,1)=1(2.16)b)F3(n0,n1)=F3(n1,n0).

**Figure 2.**
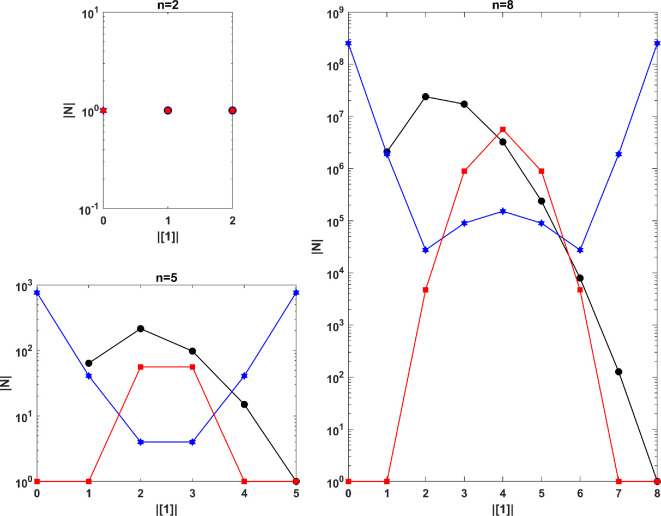
The reverse game: the number of networks corresponding to the given Nash equilibrium. The blue, black and red graphs represent the number of networks for the majority, best-shot public goods and minority games, respectively, based on the number of players adopting strategy 1.

We also determined the number of networks corresponding to the considered Nash equilibrium for *two-, five- and eight-player* games, where strategies 0 and 1 are selected by various combinations within these games. The results are presented in figure 2.

The graphs for the majority and minority games are symmetric. However, the key difference is that the highest number of networks in the minority game occurs when half of the players adopt strategy 1 and the other half adopt strategy 0. In contrast, the majority game sees the largest number of networks when all players adopt the same strategy. The graph for the best-shot public goods game is not symmetric, and its peak number of networks corresponds to a scenario where only a few players adopt strategy 1.

### 2.5. The probability of occurrence of acceptable networks

**Figure 3.**
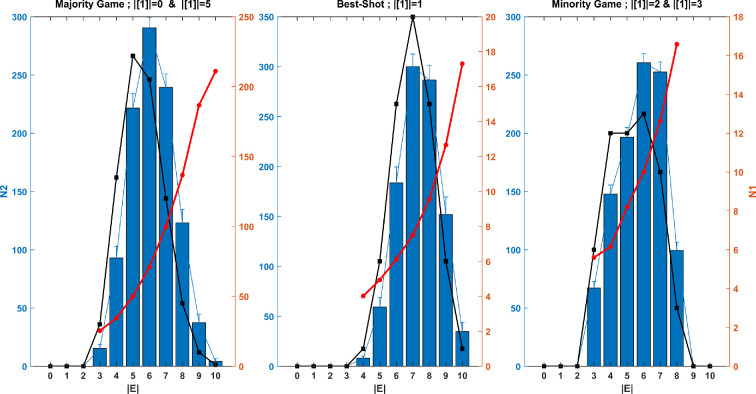
The reverse game: the probability of occurrence of each of the acceptable networks corresponding to the given Nash equilibrium. The number of networks available for the specified equilibrium is represented according to the number of edges in the black chart (column *N*1). The total number of networks that converged to the first acceptable network for the given equilibrium, achieved by randomly adding or removing edges (based on 100 independent realizations), is shown in the histogram chart (column *N*2). The ratio of converged networks to acceptable networks is illustrated in the red chart (column *N*1), calculated as follows: for the majority game, it is 50 * $\frac{N2}{N1}$; for the best-shot public goods game and minority game, it is 0.5 * $\frac{N2}{N1}.$

As illustrated in figure 2, the number of acceptable networks corresponding to the given Nash equilibrium is quite substantial. Below, we examine the distribution of these acceptable networks and the probabilities of their occurrence. The results according to the number of network edges in the *five-player* games are presented in figure 3. To calculate the probability of each acceptable network’s occurrence, we considered the space of all possible networks and randomly added or removed edges to converge towards the first acceptable network. In this simulation, the number of players adopting strategy 1 is zero or five in the majority game, one in the best-shot public goods game, and two or three in the minority game. The possible networks can have between 0 and 10 edges, with a total of 1024 possible networks. Figure 3 displays the distribution of acceptable networks based on the number of edges and the count of networks that have converged to the first acceptable network for the desired Nash equilibrium. We also calculated the ratio of converged networks to acceptable networks. The results indicate that while the distribution of acceptable networks follows a normal distribution, their probabilities of occurrence are non-uniform, increasing with density. In other words, a greater number of networks from the total space of possible networks have converged to acceptable dense networks.

To enhance understanding and facilitate a thorough assessment of the statistical claims, we have included detailed information about the simulation parameters and results of figures 2 and 3 in tables 1 and 2 of the appendix, respectively.

## 3. Discussion

Game theory uses mathematical tools to model strategic scenarios in which a player’s decisions are influenced by the choices of others. These situations arise when a player’s outcome is not solely based on their own performance but also on the performance of those around them.

Modelling multi-player games through networks allows us to concentrate on a player’s neighbours, as defined by the network edges, rather than assessing how every player’s decision affects each individual’s strategy choice. Consequently, when establishing a network among players, all vertices that influence one another must be connected by edges.

We analysed three multi-player games modelled through networks: the majority game, the minority game and the best-shot public goods game. In these games, a player’s payoff within the network either increases or decreases based on how many neighbouring vertices adopt a similar strategy. If a player’s payoff increases when more neighbours adopt the same strategy, the game is classified as one of the strategic complements (as observed in the majority game or the best-shot public goods game). Otherwise, the game is considered one of the strategic substitutes (as seen in the minority game). For each of these games, we presented the characteristics and constraints of the network between players that could create the desired Nash equilibrium. We also determined the conditions under which the desired Nash equilibrium would not be achieved under any mechanism. The networks obtained were not unique, and simulation results confirmed that the number of networks that could create the considered Nash equilibrium increased with both the number of players and the arrangement of strategies. In the best-shot public goods game, we provided a formula to calculate the number of networks based on player count and strategy arrangement. Additionally, we established limit conditions for the majority and minority games and presented a rigorous mathematical proof grounded in combinatorial principles.

While this paper demonstrates reverse game analysis for three specific games, the methodology is not inherently restricted to these examples. The approach can be applied to any game of arbitrary complexity where a well-defined network structure exists between players and that network structure is consistent with a given Nash equilibrium. This principle remains valid even for games with continuous strategy spaces, provided a well-defined condition can be established: the assumed Nash equilibrium necessitates the proposed network structure, and conversely, the players’ strategies within the resulting network constitute the Nash equilibrium of the game.

As the number of players increases, the number of networks associated with each Nash equilibrium also rises significantly. These acceptable networks can vary in density, depending on the type of game and strategy arrangement, ranging from sparse to dense configurations. The results suggest that the distribution of these networks by density follows a normal distribution. An important question is whether the occurrence probability of acceptable networks is uniform. In other words, do all these networks have an equal likelihood of existing when we consider various microscopic states that represent the network structure among players for a given Nash equilibrium, which reflects the macroscopic state of players’ strategies? We calculated the probability of each acceptable network’s occurrence by introducing random changes within the state space of all possible networks. The simulation results indicated that a larger number of networks converged to acceptable configurations with a high number of edges from the total space of possible networks. This suggests that high-density networks are more likely to occur than low-density ones. Since the likelihood of any acceptable network occurring according to the desired Nash equilibrium corresponds to the probability of that equilibrium within the network, we can conclude that the desired Nash equilibrium is more likely to manifest in acceptable dense networks. In other words, if $G_{1}$ and $G_{2}$ are acceptable networks corresponding to the Nash equilibrium ℵ, and network $G_{1}$ is denser, then the likelihood of $G_{1}$ occurring is greater than that of $G_{2}$. Consequently, the probability of Nash equilibrium ℵ in network $G_{1}$ is higher than in network $G_{2}$.

## 4. Material and methods

In §3, we present the communication rules among the vertices of acceptable networks in Nash equilibrium for each of the three games: the best-shot public goods game, the majority game and the minority game. For each of these games, within the set of all possible networks among *n* players, achieving an acceptable structure requires consideration of constraints across three groups of connections: those between players who selected strategy 0 (denoted as $V_{0}$), those between players who selected strategy 1 (denoted as $V_{1}$), and the connections between $V_{0}$ and $V_{1}$. The number of acceptable networks in the best-shot public goods game, based on the sizes of $V_{0}$ and $V_{1}$ in Nash equilibrium, is outlined in theorem 1. Subsequently, we provide a proof of this theorem utilizing combinatorial rules, particularly the multiplication principle and the principle of inclusion–exclusion.

*Proof of theorem* 1. Let G=(V,E) be an acceptable network corresponding to the given Nash equilibrium. The network *G* consists of three distinct components: (a) the inductive sub-network associated with $V_{1}$, (b) the inductive sub-network associated with $V_{0}$, and (c) the sub-network representing the connections between the vertices in $V_{1}$ and $V_{0}$. First, we calculate the number of possible sub-networks for each component, and then we determine the total number of networks using the multiplication principle:

(a) The inductive sub-network corresponding to $V_{1}$ must have no edges. Therefore, this component can exist in only one configuration.
(b) The inductive sub-network corresponding to $V_{0}$ contains $n_{0}$ vertices. There are no restrictions on the presence or absence of edges in this part, so all possible structures must be considered. The maximum number of possible edges in this component is given by $\frac{n_{0}(n_{0}-1)}{2}$. Consequently, the number of inductive sub-networks corresponding to $V_{0}$ is $2^{\frac{n_{0}\left(n_{0}-1\right)}{2}}$.
(c) To calculate the number of acceptable sub-networks corresponding to connections between vertices in $V_{1}$ and $V_{0}$, we apply the rule that a vertex in $V_{0}$ must have at least one neighbour in $V_{1}$. The maximum number of possible edges in this component is $n_{0}\times n_{1}$, leading to a maximum number of structures equal to $2^{n_{0}\times n_{1}}$. However, not all these structures are acceptable; only those configurations where every vertex in $V_{0}$ has at least one edge connecting it to a vertex in $V_{1}$ should be counted. Thus, we need to exclude structures where no vertex from $V_{0}$ has a neighbour in $V_{1}$. The number of configurations where a specific vertex in $V_{0}$ lacks neighbours in $V_{1}$ is given by $2^{(n_{0}-1)\times n_{1}}$. Therefore, the count of structures where exactly one unspecified vertex has no neighbours in $V_{1}$ is equal to $n_{0}\times 2^{(n_{0}-1)\times n_{1}}$. Similarly, for each integer k such that $1\leq k\leq n_{0}$, the number of configurations where k specified (or unspecified) vertices from $V_{1}$ have no neighbours in $V_{0}$ is expressed as: $2^{(n_{0}-k)\times n_{1}}$ (n0k×2(n0-k)×n1). According to the principle of inclusion–exclusion, the total number of acceptable sub-networks for this component is ∑i=0n0(-1)i×n0i×2n0-i×n1.

Finally, using the multiplication principle, the overall number of acceptable networks is equal to the product of the counts from these three components, resulting in the formula presented.∎

In contrast to the best-shot public goods game, which enforces a strict requirement that every vertex in $V_{0}$ must have at least one neighbour in $V_{1}$, the rules in the majority and minority games are more flexible. In the majority game, the number of connections between a vertex in $V_{0}$ and a vertex in $V_{1}$ must be fewer than the connections between two vertices either in $V_{0}$ or in $V_{1}$. Conversely, in the minority game, this number must be greater. As a result, a general function for calculating the number of acceptable networks is not readily available. However, in theorem 2 and theorem 3, we determine the number of these networks under specific conditions for these two games, as demonstrated below.

*Proof of theorem* 2. (a) The strategy for all vertices in the network is 0 only if each vertex has a degree of at least 1. The maximum number of possible edges among $n_{0}$ vertices is given by $\frac{n_{0}(n_{0}-1)}{2}$ , which means the total number of potential networks is equal to $2^{\frac{n_{0}(n_{0}-1)}{2}}$. Similar to the reasoning used in proving theorem 1, we can apply the principle of inclusion–exclusion to determine that the number of networks with $n_{0}$ vertices, where each vertex has a degree of at least 1, is given by ∑i=0n0(-1)i×n0i×2n0-i2.

(b) Since the vertices in sets $V_{0}$ and $V_{1}$ are subject to the same conditions and rules, the equality is clearly preserved.

(c) If all vertices, except for one, adopt strategy 0, then the vertex that adopts strategy 1 must have a degree of 0. Consequently, the number of acceptable networks corresponds to the scenario in which all vertices in a network with $n_{0}$ vertices choose strategy 0. Additionally, in a network with $n_{0}+2$ vertices, where all but two vertices select strategy 0, the two vertices that adopt strategy 1 must be connected to each other rather than to any other vertices. As a result, no additional structures are formed, and once again, the number of configurations is equal to the number of networks with $n_{0}$ vertices where all vertices choose strategy 0.∎

*Proof of theorem 3*. The proof is similar to parts (c) and (b) of theorem 2, respectively.∎

## Data Availability

Data is available online [57].

## A. Appendix

See tables 1 and 2.

**Table 1.** The number of acceptable networks for each game.

number of nodes	game	number of players adopting strategy 1								
		0	1	2	3	4	5	6	7	8
*n* = 2	*majority*	1	1	1
	*best-shot*	0	1	1
	*minority*	1	1	1
*n* = 5	*majority*	768	41	4	4	41	768
	*best-shot*	0	64	216	98	15	1
	*minority*	1	1	56	56	1	1
*n* = 8	*majority*	252 522 488	1 887 278	27 454	89 792	152 219	89 792	27 454	1 887 278	252 522 488
	*best-shot*	0	2 097 152	23 887 872	17 210 368	3 240 000	238 328	7938	127	1
	*minority*	1	1	4700	890 534	5 651 384	890 534	4700	1	1

**Table 2.** The probability of occurrence for each of the acceptable networks. ***f***: The number of networks available for the specified Nash equilibrium. ***G***: The total number of networks that converge to the first acceptable network through random addition or removal of edges (based on 100 independent realizations). ***h***: Error bar.

game	conditions		number of edges										
			0	1	2	3	4	5	6	7	8	9	10
*majority game*	$\begin{array}{r} \left|\left[1\right]\right|=0\\ \text{or}\;|[1]|=5 \end{array}$	*f*	0	0	0	30	135	222	205	120	45	10	1
		*g*	0	0	0	15.25	92.96	221.58	290.36	239.29	123.01	37.33	4.22
		*h*	0	0	0	3.36	9.89	12.59	9.01	11.61	11.69	7.31	2.27
*best-shot*	$\left|\left[1\right]\right|=1$	*f*	0	0	0	0	1	6	15	20	15	6	1
		*g*	0	0	0	0	8.04	59.24	183.58	300.06	286.58	151.88	34.62
		*h*	0	0	0	0	2.17	9.54	16.09	12.56	14.74	17.45	9.36
*minority game*	$\begin{array}{r} \left|\left[1\right]\right|=2\\ \text{or}\;|[1]|=3 \end{array}$	*f*	0	0	0	6	12	12	13	10	3	0	0
		*g*	0	0	0	67.15	147.77	196.52	260.46	252.62	99.48	0	0
		*h*	0	0	0	5.67	7.69	8.57	7.76	8.49	6.90	0	0
